# Supplementary material for: Acute Respiratory Distress Syndrome and Time to Weaning Off the Invasive Mechanical Ventilator among Patients with COVID-19 Pneumonia
Source: J Clin Med. 2021 Jun 30;10(13):2935. doi: 10.3390/jcm10132935 (PMC8269065; doi:10.3390/jcm10132935)
Supplement: Supplementary file 1 [file jcm-10-02935-s001.zip › jcm-1278590-supplementary.pdf]

Supplementary Material

Human Subjects Protection

The study was approved by the Institutional Review Board (IRB) at the University of Louisville Human Subjects Research Protection Program Office (IRB number 20.0257) and by the research offices at each participating hospital. The study was exempt from informed consent.

Data Collection

Data were abstracted from hospital electronic medical records using Research Electronic Data Capture software (REDCap version 10.1.0). The collected data included patient age, sex, race/ethnicity, body mass index, residence, medical and social history, physical examination findings, laboratory findings, chest radiographs and chest computerized tomography (CT) findings, medications, intensive care unit (ICU) admission, and need for mechanical ventilation. Race was categorized as Black, White, and other. Ethnic group was categorized as Hispanic or non-Hispanic.

Study Coordinating Center

The Center of Excellence for Research in Infectious Diseases (CERID), located at the University of Louisville Division of Infectious Diseases, directed all study operations (11). Members of CERID developed the study data collection form and the study database, collected data from hospital electronic medical records, recorded data in REDCap and performed quality control on all collected data. Once all data queries were resolved, the study database was locked for data analysis.

Supplementary Table S1. Univariate Analysis of Risk Factors Associated with the Weaning off the Invasive Mechanical Ventilation.

	Odds Ratio	Lower	Upper	*p* value
Age (years)	0.99	0.98	1.00	0.190
Male sex	0.54	0.31	0.94	0.028
White race	1.07	0.62	1.85	0.805
Black race	0.76	0.45	1.28	0.303
Hispanic ethnicity	0.85	0.55	1.32	0.465
Neoplastic disease	0.81	0.25	2.66	0.734
Renal disease	0.73	0.36	1.49	0.387
Heart failure	0.56	0.33	0.95	0.031
Coronary artery disease	0.47	0.24	0.91	0.025
COPD	0.75	0.25	2.24	0.608
Cerebrovascular disease	0.59	0.39	0.89	0.012
Smoking history (former or current)	0.79	0.42	1.51	0.484
Diabetes	0.54	0.29	0.98	0.044
Obesity	0.74	0.44	1.25	0.261
Hypertension	0.73	0.52	1.03	0.076
Hyperlipidemia	0.89	0.58	1.35	0.579
Temperature	0.85	0.63	1.14	0.276
Respiratory rate	0.99	0.77	1.26	0.912

Mean arterial pressure	1.12	0.90	1.39	0.313
Heart rate (1 SD increase)	1.00	0.75	1.32	0.993
Blood urea nitrogen (1 SD increase)	0.75	0.53	1.08	0.120
Glucose (1 SD increase)	0.70	0.49	1.00	0.048
Hematocrit (1 SD increase)	1.10	0.89	1.36	0.380
Sodium (1 SD increase)	0.93	0.80	1.09	0.375
IL-6 > 65 pg/mL	0.47	0.20	1.07	0.073
CRP > 150 (mg/L)	0.78	0.48	1.26	0.309
D-Dimer > 1800 ng/mL	0.78	0.53	1.13	0.188
Ferritin > 650 (ng/mL)	0.63	0.37	1.09	0.100
Lactate > 2 mmol/L	0.54	0.34	0.86	0.009
Altered mental status	0.74	0.30	1.83	0.514
Pleural effusion	0.53	0.22	1.26	0.151
Severe ARDS	0.52	0.37	0.72	<0.001

Abbreviations: ARDS, acute respiratory distress syndrome; COPD, chronic obstructive pulmonary disease; CRP, C-reactive protein; IL-6, interleukin-6; SD, standard deviation.

Supplementary Table S2. Univariate Analysis of Risk Factors Associated with Hospital Mortality.

	**Odds Ratio**	**Lower**	**Upper**	***p*-value**
Age (years)	1.03	1.00	1.05	0.032
Male sex	2.22	1.14	4.32	0.019
White race	1.14	0.73	1.77	0.574
Black race	0.97	0.63	1.49	0.889
Hispanic ethnicity	0.99	0.50	1.97	0.983
Neoplastic disease	1.57	0.48	5.10	0.453
Renal disease	1.10	0.50	2.43	0.809
Heart failure	1.21	0.39	3.73	0.736
Coronary artery disease	2.11	1.03	4.34	0.042
COPD	1.42	0.60	3.34	0.425
Cerebrovascular disease	2.12	1.15	3.89	0.016
Smoking history (former or current)	1.14	0.65	2.01	0.640
Diabetes	1.69	0.77	3.74	0.193
Obesity	0.92	0.43	1.99	0.832
Hypertension	2.14	1.66	2.76	<0.001
Hyperlipidemia	1.21	0.60	2.44	0.586
Temperature	1.14	0.86	1.51	0.362
Respiratory rate	1.14	0.95	1.35	0.150
Mean arterial pressure	0.85	0.64	1.14	0.291
Heart rate (1 SD increase)	1.06	0.63	1.78	0.828
Blood urea nitrogen (1 SD increase)	1.31	1.13	1.52	<0.
Glucose (1 SD increase)	1.43	1.17	1.76	0.001
Hematocrit (1 SD increase)	1.11	0.76	1.61	0.590
Sodium (1 SD increase)	1.42	1.04	1.94	0.026
IL-6 > 65 pg/mL	1.67	0.81	3.44	0.163
CRP > 150 (mg/L)	1.14	0.65	1.99	0.643
D-Dimer > 1800 ng/mL	1.70	1.18	2.46	0.005
Ferritin > 650 (ng/mL)	1.79	0.87	3.68	0.114
Lactate > 2 mmol/L	2.91	1.54	5.49	0.001
Altered mental status	3.03	1.51	6.10	0.002
Pleural effusion	2.55	1.47	4.43	0.001
Severe ARDS	2.22	1.32	3.76	0.003

Abbreviations: ARDS, acute respiratory distress syndrome; COPD, chronic obstructive pulmonary disease; CRP, C-reactive protein; IL-6, interleukin-6; SD, standard deviation.

Supplementary Table S3. Severity of Disease Among All Patients Hospitalized with COVID-19 Pneumonia.

	**Critical Illness n = 219**	**Non-critical Illness n = 303**	***p* value**
Severity of Disease n (%)			
Altered mental status	56 (26)	25 (8)	<0.001
Pleural effusion	31 (14)	38 (13)	0.684
Degree of hypoxemia at time of critical illness			<0.001
Normal	66 (30)	303 (100)	
Mild	44 (20)	0 (0)	
Moderate	52 (24)	0 (0)	
Severe	57 (26)	0 (0)	
Pneumonia Severity Index Risk Class (%)			<0.001
Risk Class I	23 (11)	65 (21)	
Risk Class II	47 (21)	108 (36)	
Risk Class III	44 (20)	48 (16)	
Risk Class IV	68 (31)	59 (19)	
Risk Class V	37 (17)	23 (8)	
qSOFA score (%)			<0.001
0	38 (17)	154 (51)	
1	102 (47)	121 (40)	
2	53 (24)	27 (9)	
3	26 (12)	1 (0)	
CURB-65 Score (%)			0.001
0	51 (23)	101 (34)	
1	57 (26)	97 (32)	
2	54 (25)	59 (20)	
3	35 (16)	34 (11)	
4	18 (8)	8 (3)	
5	3 (1)	0 (0)	

Patients with advanced directives were not included; Abbreviations: COVID-19: coronavirus disease 2019; CURB-65: confusion, uremia, respiratory rate, BP, age ≥65 years; qSofa: quick sequential organ failure assessment.
